# Vitamin D stimulates placental L-type amino acid transporter 1 (LAT1) in preeclampsia

**DOI:** 10.1038/s41598-022-08641-y

**Published:** 2022-03-17

**Authors:** Xiaotong Jia, Yang Cao, Lingyu Ye, Xueqing Liu, Yujia Huang, Xiaolei Yuan, Chunmei Lu, Jie Xu, Hui Zhu

**Affiliations:** 1 Department of Physiology, Harbin Medical University, Harbin, 150081 China; 2 Department of Obstetrics and Gynecology, Second Affiliated Hospital of Harbin Medical University, Harbin, 150081 China

**Keywords:** Intrauterine growth, Calcium and vitamin D

## Abstract

Vitamin D insufficiency/deficiency has been linked to an increased risk of preeclampsia. Impaired placental amino acid transport is suggested to contribute to abnormal fetal intrauterine growth in pregnancies complicated by preeclampsia. However, if vitamin D-regulated amino acid transporter is involved in the pathophysiologic mechanism of preeclampsia has not been clarified yet. The aberrant expression of key isoform of L-type amino acid transporter LAT1 was determined by western blot and immunohistochemistry in the placenta from normotensive and preeclamptic pregnancies. The role for vitamin D on placental LAT1 expression was investigated through the exposure of HTR-8/SVneo human trophoblast cells to the biologically active 1,25(OH)_2_D_3_ and the oxidative stress-inducer cobalt chloride (CoCl_2_). Our results showed that placental LAT1 expression was reduced in women with preeclampsia compared to normotensive pregnancies, which was associated with decreased expression of vitamin D receptor (VDR). 1,25(OH)_2_D_3_ significantly upregulated LAT1 expression in placental trophoblasts, and also prevented the decrease of mTOR activity under CoCl_2_-induced oxidative stress. siRNA targeting VDR significantly attenuated 1,25(OH)_2_D_3_-stimulated LAT1 expression and mTOR signaling activity. Moreover, treatment of rapamycin specifically inhibited the activity of mTOR signaling and resulted in decrease of LAT1 expression. In conclusion, LAT1 expression was downregulated in the placenta from women with preeclampsia. 1,25(OH)_2_D_3_/VDR could stimulate LAT1 expression, which was likely mediated by mTOR signaling in placental trophoblasts. Regulation on placental amino acid transport may be one of the mechanisms by which vitamin D affects fetal growth in preeclampsia.

## Introduction

Preeclampsia (PE) is a pregnancy-specific hypertensive disorder which is associated with substantial morbidity and mortality in mothers and their fetuses worldwide^1^. It also increases the future risk of metabolic and cardiovascular disease later in life^2^. The pathogenesis of preeclampsia remains not fully elucidated, but the impaired remodeling of uterine spiral artery is believed at the root etiology of this disease^3^. This causes reduced blood flow to the placenta and leads to an exposure of the developing fetus to an insufficient oxygen and nutrient supply, which contributes to fetal growth restriction in preeclampsia.

Placental trophoblastic transport of amino acids is vital for the growing fetus in protein synthesis, metabolic and biosynthetic processes. Decreased trophoblastic transfer of amino acids is believed to contribute to fetal growth restriction during pregnancy^4,5^. The L-type amino acid transporter (LAT) is a primary transport system present in the placenta, which is required for the Na^+^-independent antiport of essential amino acids. The isoform LAT1, as a major accumulative transporter in L-type system, is abundant on the apical surface of syncytiotrophoblast and active in the uptake of specific amino acids from maternal circulation into the placenta^6^. It has been found that trophoblastic LAT1 expression is increased in women with type 2 diabetes mellitus and associated with birth weight and neonatal fat mass^7^, but decreased in the placentas from small-for gestational-age (SGA) fetus^8^. In addition, inhibition of LAT1 transporters is found to be involved in the mouse implantation and placentation by affecting trophoblast differentiation and invasion^9,10^. Placental transport of amino acids is intensively regulated by mammalian target of rapamycin (mTOR) signaling via modulating translocation or global expression of amino acid transporters in trophoblast cells^11^. Jansson et al. demonstrated that mTOR mediated amino acid uptake by regulating transporter cell surface abundance in primary human trophoblast cells^12^. We previously reported that suppression of mTOR signaling down-regulated the expressions of LAT1 and LAT2 in placental trophoblasts^13^. So far, there are few published data regarding the expression and regulatory mechanism of LAT1 in the placenta from preeclampsia.

Emerging evidence have shown that insufficient or deficient vitamin D status in pregnancy increases the risk of several pregnancy complications including preeclampsia, fetal growth restriction, and preterm birth^14^. On the contrary, supplementation of vitamin D during pregnancy has been suggested to reduce the risk of preeclampsia^15^. Although the role for vitamin D in placental trophoblasts has not been fully established, it has been clearly demonstrated that vitamin D exerted anti-inflammatory and anti-oxidative stress properties in placental trophoblasts. In our previous studies, we found that the biologically active 1,25(OH)_2_D_3_ significantly suppressed the cyclooxygenase-2 activity and the downstream prostaglandin E_2_ production in placental trophoblasts in response to hypoxic stimulation^16^, it also attenuated the oxidative stress-induced microparticle shedding from placental trophoblasts^17^. Vitamin D is also implicated to involve in trophoblast proliferation and migration^18^, activation of autophagy^19^, alleviation of insulin resistance^20^ in placental trophoblasts. However, there was little data except one study from Southampton Women’s survey showed that maternal 25(OH)D_3_ and vitamin D binding protein (VDBP) levels were positively related to expression of specific placental amino acid transporters, indicating that vitamin D may be involved in the regulation of placental amino acid transport^21^.

To further study the beneficial properties of vitamin D in placental trophoblasts, we aimed to investigate if vitamin D-regulated amino acid transporter was involved in the pathophysiology of preeclampsia in the present study. To test this, the expression of LAT1 was determined in human placentas from preeclampsia. Then, the role for vitamin D in placental LAT1 expression and underlying mechanisms was investigated through the exposure of HTR-8/SVneo human placental trophoblast cells to 1,25(OH)_2_D_3_ and the hypoxia mimic CoCl_2_.

## Results

### Reduced placental VDR expression is associated with decreased expression of LAT1 in preeclampsia

**Figure 1:**
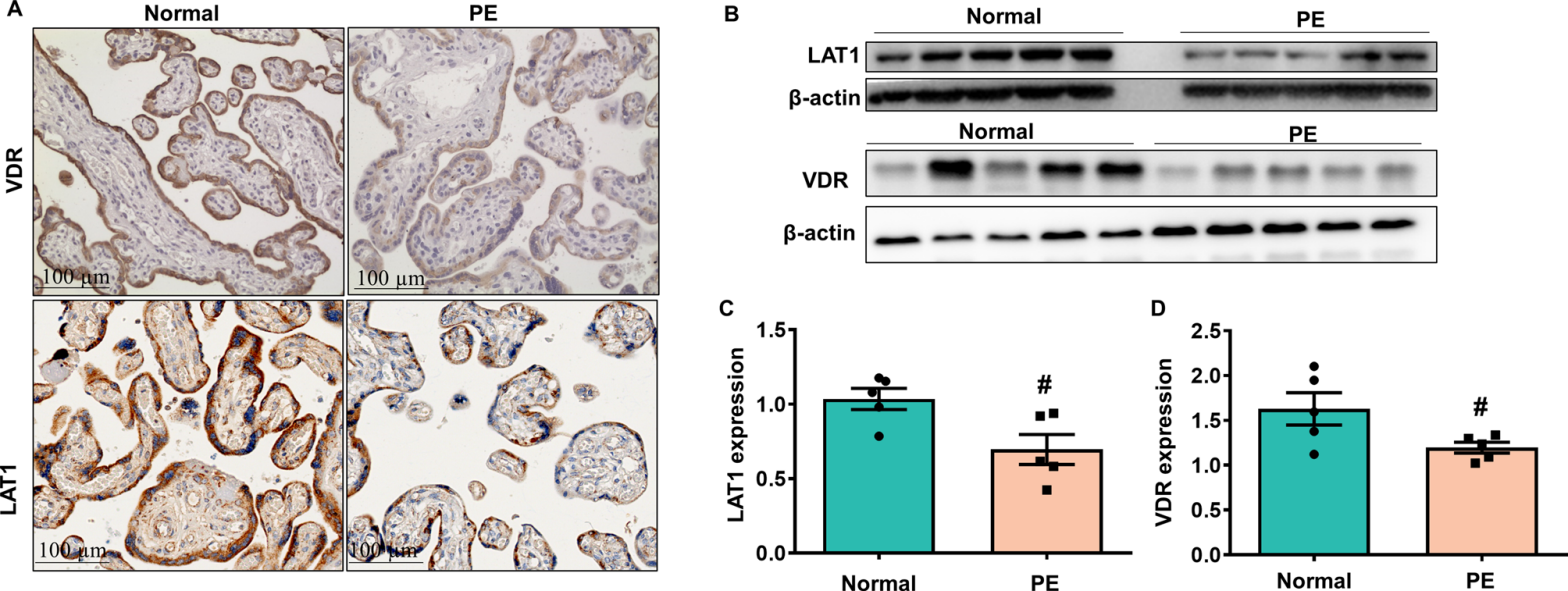
Expression of LAT1 and VDR in normal and preeclamptic placentas. (**A**) Representative immunostaining images of LAT1 and VDR expressions in tissue sections from normal and preeclamptic (PE) placentas. Bar = 100 micron. (**B**) Representative blots of LAT1 and VDR expression in placenta from normotensive and PE pregnancies. (**C,D**) The bar graphs show the relative density of protein expression for LAT1 and VDR after normalization with β-actin expression in each sample. n = 5, ^#^*P* < 0.05, PE vs Normal.

**Figure 2:**
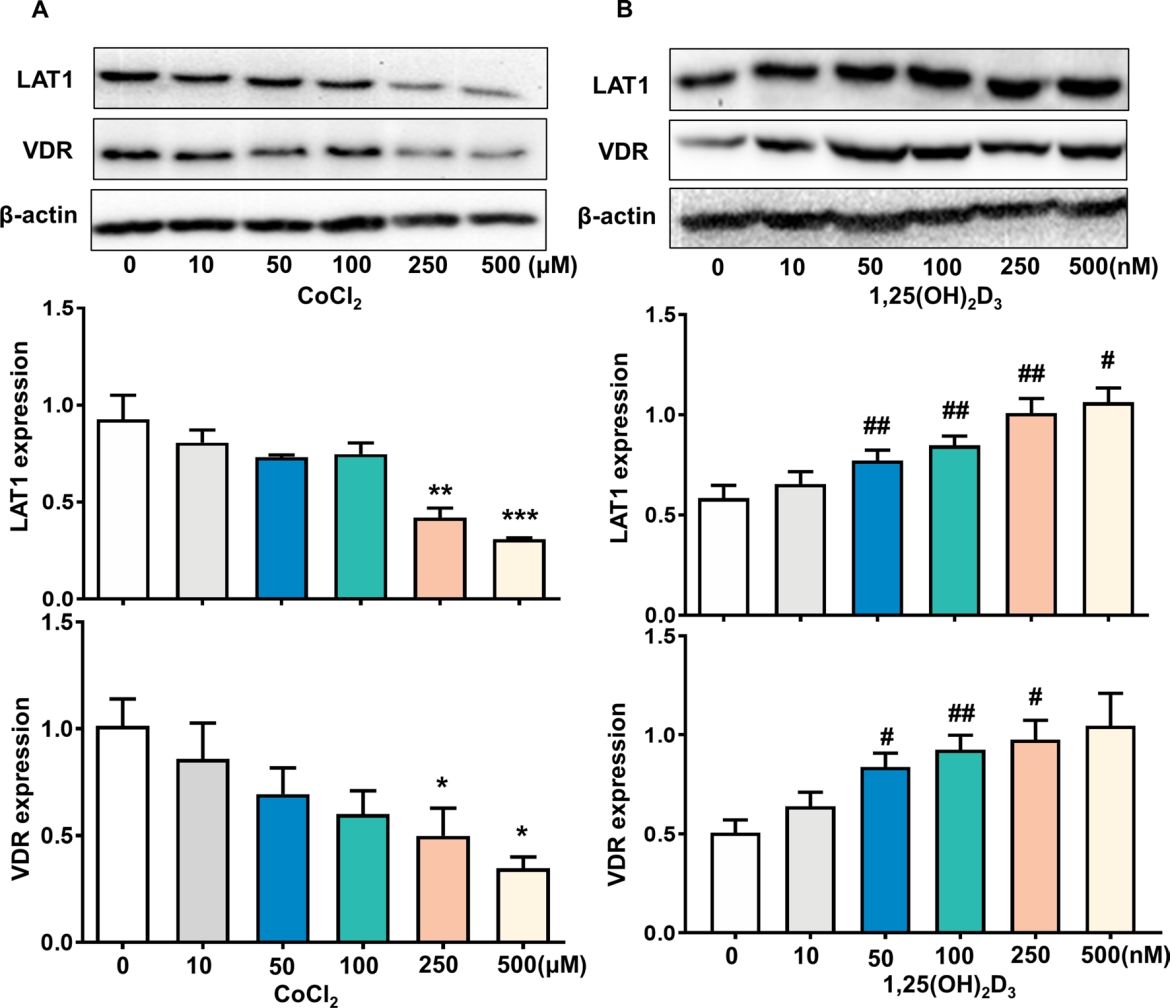
Effects of 1,25(OH)_2_D_3_ and CoCl_2_ on LAT1 and VDR expression in placental trophoblasts. (**A**) Protein expression for LAT1 and VDR in HTR-8/SVneo trophoblast cells treated with different concentrations of CoCl_2_. The bar graphs show relative protein expression for LAT1 and VDR after normalized against β-actin in each sample from three independent experiments. CoCl_2_ induced a dose-dependent decrease in both LAT1 and VDR expression in trophoblasts. *P < 0.05, **P < 0.01, ***P < 0.001, CoCl_2_ treated vs control cells. (**B**) Protein expression for LAT1 and VDR in HTR-8/SVneo trophoblast cells treated with different concentrations of 1,25(OH)_2_D_3_. The bar graphs show relative protein expression for LAT1 and VDR after normalization with β-actin in each sample from four independent experiments. In contrast to CoCl_2_, 1,25(OH)_2_D_3_ stimulated LAT1 and VDR expression in a dose-dependent manner in trophoblast cells. ^#^*P* < 0.05, ^##^*P* < 0.01, 1,25(OH)_2_D_3_ treated vs control cells.

To explore the involvement of LAT1 in preeclampsia, we first observed the expression of LAT1 in placenta from women with preeclampsia. As shown in Fig. 1A, expressions of LAT1 and VDR were mainly located in the cytoplasm of placental syncytiotrophoblasts. The LAT1 expression was significantly reduced in the placenta from preeclamptic pregnancies compared with that from normotensive pregnancies (Fig. 1B,C). The VDR expression was also decreased in preeclamptic placentas (Fig. 1B,D). Since placental hypoxia is at the root pathology of preeclampsia, we further determined the effects of hypoxia on LAT1 expression in cultured human HTR-8/SVneo trophoblast cells. Our results showed that the expression of LAT1 was dose-dependently decreased when trophoblast cells were cultured with an increasing concentration of CoCl_2_ compared to untreated cells (Fig. 2A). Similar to the effects of CoCl_2_ on LAT1, the VDR expression was also reduced in a dose-dependent manner in trophoblast cells treated with CoCl_2_ (Fig. 2A).

### 1,25(OH)_2_D_3_ Stimulates LAT1 expression and prevents hypoxia-induced decrease of LAT1 expression in placental trophoblasts

**Figure 3:**
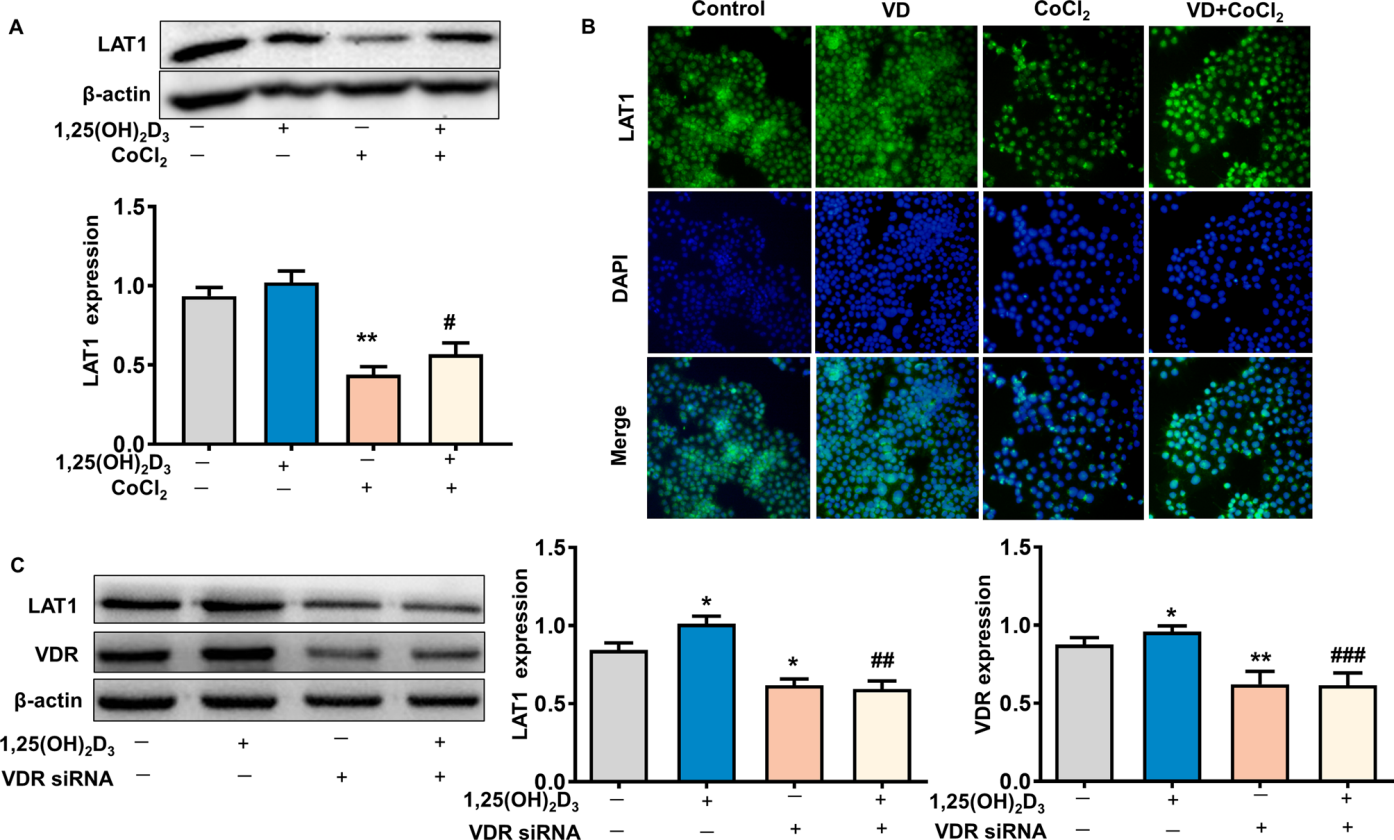
Effects of 1,25(OH)_2_D_3_ on CoCl_2_-induced LAT1 expression in placental trophoblasts. (**A**) Protein expression of LAT1 detected by Western blot in HTR-8/SVneo trophoblast cells treated with CoCl_2_ in the presence or absence of 1,25(OH)_2_D_3_. The decreased LAT1 expression induced by CoCl_2_ was attenuated when cells were treated with 1,25(OH)_2_D_3_. The bar graphs show relative protein expression after being normalized by β-actin in each sample from six independent experiments. **P < 0.01, CoCl_2_ treated vs control; ^#^P < 0.05, 1,25(OH)_2_D_3_ + CoCl_2_ vs CoCl_2_ alone. (**B**) Representative imaging of immunofluorescent staining of LAT1 in trophoblasts treated by CoCl_2_ with or without 1,25(OH)_2_D_3_. Consistent with Western blot results, 1,25(OH)_2_D_3_ could prevent CoCl_2_-induced decreased LAT1 expression in trophoblast cells. (**C**) Protein expression of VDR and LAT1 expression in trophoblasts treated with VDR siRNA in the presence or absence of 1,25(OH)_2_D_3_. Inhibition of VDR expression attenuates 1,25(OH)_2_D_3_-stimulated LAT1 expression in trophoblast cells. The bar graphs show relative VDR and LAT1 expression after being normalized by β-actin in each sample from six independent experiments, *P < 0.05, **P < 0.01, treated vs control; ^##^P < 0.01, ^###^P < 0.001, 1,25(OH)_2_D_3_ + VDR siRNA vs 1,25(OH)_2_D_3_ alone.

The role for vitamin D on LAT1 expression in placental trophoblasts was investigated using cultured HTR-8/SVneo trophoblast cells which were cultured with 1,25(OH)_2_D_3_ in the presence or absence of CoCl_2_. The results showed that 1,25(OH)_2_D_3_ treatment could stimulate both LAT1 and VDR expression in a dose-dependent way in trophoblast cells (Fig. 2B). Moreover, the LAT1 expression was significantly reduced in the cells cultured with 250 μM of CoCl_2_, and such a CoCl_2_-induced decrease of LAT1 was significantly prevented when the cells were treated with 100 nM of 1,25(OH)_2_D_3_ (Fig. 3A). The LAT1 expression was also examined by immunofluorecent staining in trophoblast cells. Consistent with the Western blot data, 1,25(OH)_2_D_3_ could attenuate CoCl_2_-induced decrease of LAT1 expression in trophoblast cells (Fig. 3B).

Then, VDR siRNA silencing was performed to knockdown VDR expression to elucidate the specificity of vitamin D-stimulated LAT1 in trophoblast cells. Our results showed that VDR expression was significantly reduced in the cells transfected with VDR siRNA compared with control cells, and 1,25(OH)_2_D_3_ was unable to upregulate VDR expression when the cells were treated with VDR siRNA (Fig. 3C). The LAT1 expression was markedly downregulated in the cells transfected with VDR siRNA, and 1,25(OH)_2_D_3_ could not upregulate LAT1 expression when the VDR expression was knocked down in trophoblast cells (Fig. 3C).

### 1,25(OH)_2_D_3_ Upregulates mTOR signaling via VDR in placental trophoblasts

**Figure 4:**
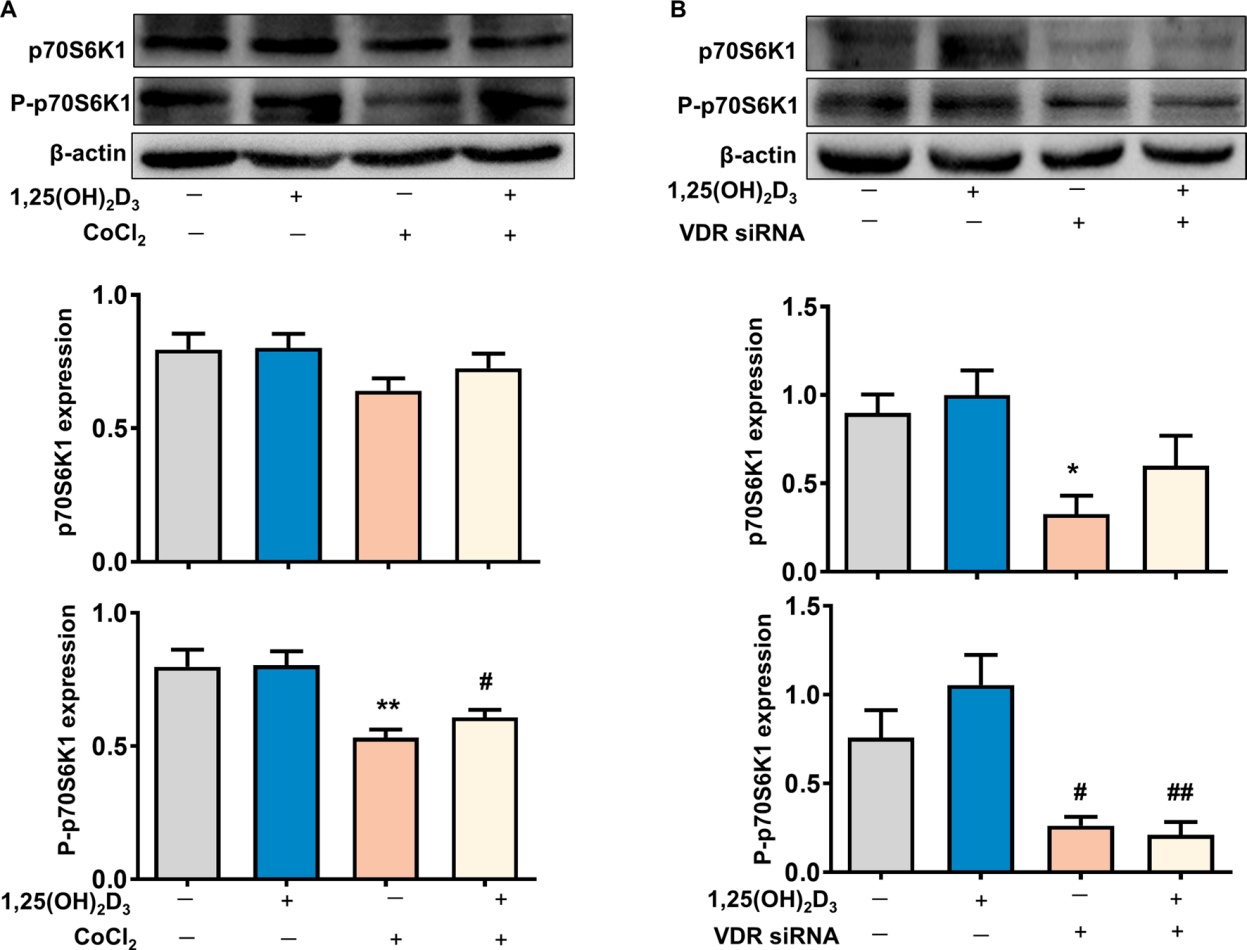
Effects of 1,25(OH)_2_D_3_ on altered mTOR activity induced by CoCl_2_ in placental trophoblasts. (**A**) Protein expression for p70S6K1 and P-p70S6K1 in HTR-8/SVneo trophoblast cells treated with CoCl_2_ in the presence or absence of 1,25(OH)_2_D_3_. p70S6K1 is one downstream indicator of mTOR signaling activity, 1,25(OH)_2_D_3_ prevented CoCl_2_-induced decrease of P-p70S6K1 expression in trophoblasts. The bar graphs show relative expression of p70S6K1 and P-p70S6K1 after being normalized by β-actin in each sample from six independent experiments. **P < 0.01, CoCl_2_ treated vs control; ^#^P < 0.05, 1,25(OH)_2_D_3_ + CoCl_2_ vs CoCl_2_ alone. (**B**) Protein expression for p70S6K1 and P-p70S6K1 in HTR-8/SVneo trophoblast cells treated with VDR siRNA in the presence or absence of 1,25(OH)_2_D_3_. Inhibition of VDR expression markedly suppressed P-p70S6K1 and total p70S6K1 expression in trophoblasts. The bar graphs show relative expression of p70S6K1 and P-p70S6K1 after being normalized by β-actin in each sample. Data are from four independent experiments. *P < 0.05, VDR siRNA vs control; ^#^P < 0.05, ^##^P < 0.01, treated vs 1,25(OH)_2_D_3_ alone.

In order to investigate whether mTOR signaling is involved in the vitamin D-regulated LAT1 expression, we determined the effect of 1,25(OH)_2_D_3_ on the activity of mTOR signaling using the mTOR downstream effector, 70 kD ribosomal protein S6 kinase 1 (p70S6K1). As shown in Fig. 4A, the expression of phosphorylated p70S6K1 (P-p70S6K1) but not total p70S6K1 was downregulated in the cells cultured with CoCl_2_, and such a CoCl_2_-induced decrease of P-p70S6K1 was attenuated by the treatment of 1,25(OH)_2_D_3_. Moreover, siRNA silence targeting VDR significantly suppressed the expressions of P-p70S6K1 and total p70S6K1 in trophoblast cells (Fig. 4B).

### Suppression of mTOR pathway downregulates LAT1 expression in placental trophoblasts

**Figure 5:**
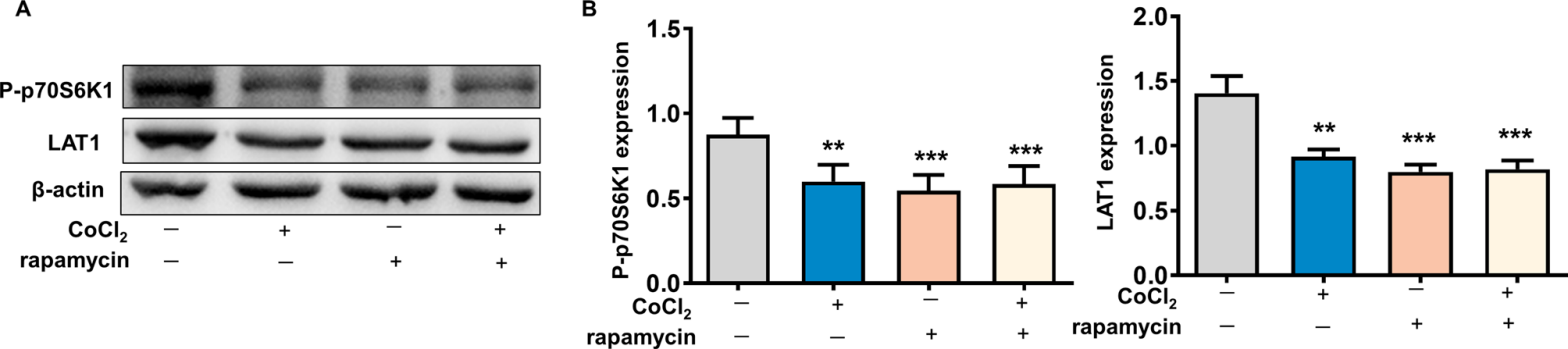
Effects of mTOR inhibitor on LAT1 expression in placental trophoblasts. (**A**) Protein expression of LAT1 in HTR-8/SVneo trophoblast cells treated with mTOR inhibitor rapamycin in the presence or absence of CoCl_2_. Rapamycin markedly inhibit P-p70S6K1 expression in trophoblast cells. The LAT1 expression was significantly downregulated in cells treated with rapamycin. (**B**) The bar graphs show relative expression of LAT1 after normalization by β-actin in each sample from six independent experiments. **P < 0.01, ***P < 0.001, treated vs control.

We used the specific inhibitor of mTOR signaling, rapamycin, to determine the critical role for mTOR signaling in the regulation of LAT1 expression in trophoblast cells. Treatment of rapamycin significantly inhibited P-p70S6K1 expression in trophoblast cells, suggesting a suppressed activity of mTOR signaling caused by rapamycin. Importantly, LAT1 expression was clearly decreased when the mTOR signaling was inhibited by rapamycin in the presence or absence of CoCl_2_ (Fig. 5). The P-p70S6K1 and LAT1 expressions were also suppressed in the cells cultured with CoCl_2_ alone.

## Material and methods

### Placenta tissue collection

Human placentas were collected immediately after delivery at the Second Affiliated Hospital of Harbin Medical University. A total of 10 placentas were used in the study, 5 from normal and 5 from preeclamptic pregnancies. Uncomplicated pregnancy is defined as pregnancy with a blood pressure < 140/90 mmHg, and absence of proteinuria, obstetrical and medical complications. Preeclampsia was defined as systolic blood pressure ≥ 140 mmHg or diastolic blood pressure ≥ 90 mmHg with at least two separate readings and coexistence of proteinuria (> 1+) at dipstick or ≥ 300 mg protein/day in urine. Patients complicated with HELLP syndrome, diabetes, and/or renal disease were excluded.

### Study approval

Human placenta collection was approved by the Ethical Committee for the Use of Human Samples of Harbin Medical University (82001577). All the participants signed a written informed consent for study enrollment. All the experiments were performed in accordance with the relevant guidelines and regulations of ethics committee of Harbin Medical University.

### Human trophoblast cell culture and treatment

The HTR-8/SVneo human trophoblast cell line was obtained from BeNa Cultrue Collection (Beijing, China). The cells were routinely maintained in Dulbecco’s Modified Eagle Media: Nutrient Mixture F-12 (DMEM/F12) medium supplemented with 10% fetal bovine serum and 1% penicillin/streptomycin and incubated under standard conditions of 5% CO_2_ at 37 °C. 1,25(OH)_2_D_3_ was used as bioactive vitamin D, and cobalt chloride (CoCl_2_) was used as an inducer of hypoxia that causes oxidative stress in placental trophoblasts. HTR-8/SVneo cells were seeded in six-well plates (4 × 10^5^ cells/well) and treated with CoCl_2_ at concentrations of 10, 50, 100, 250, and 500 μM or with 1,25(OH)_2_D_3_ at 10, 50, 100, 250, and 500 nM for 24 h. In the experiment to test the role for vitamin D on hypoxia-induced LAT1 expression, CoCl_2_ at a concentration of 250 μM and 1,25(OH)_2_D_3_ at a concentration of 100 nM were used. At the end of each experiment, total cellular protein or RNA was extracted and used to determine protein expression or mRNA expression.

### VDR siRNA transfection

VDR Knockdown was carried out by transfection of VDR siRNA in HTR-8/SVneo cells using Lipofectamine 2000 transfection reagent (Invitrogen, CA, USA) according to the manufacture’s instruction. Briefly, 18 h after seeding, cells were starved with serum-free DMEM/F12 for 2 h and then incubated with Opti-MEM medium for 6 h, which contains 50 nM of VDR siRNAs (GenePharma, Suzhou, China) mixed with Lipofectamine transfection reagent. Cellular protein was collected 48 h after transfection, and protein expression for VDR and LAT1 was then determined by Western blot.

### Immunohistochemical staining

Fresh placental tissue was fixed with 10% formalin and embedded in paraffin. Expression of LAT1 and VDR was examined by immunohistochemistry (IHC) staining of paraffin-embedded tissue sections. A standard immunohistochemistry staining procedure was performed as previously described^22^. Stained slides with the same antibody were all processed at the same time. Stained slides were reviewed under a microscope, and images were captured with a digital scanning microscopy imaging system (PreciPoint, Germany).

### Protein expression by western blot

Placental and trophoblastic protein expressions for LAT1, VDR, p70S6K1, and phospho-p70S6K1 were examined by western blot. Antibody against human LAT1 (sc-134994) was purchased from Santa Cruz Biotechnology (CA, USA), antibodies against human VDR (AF6159), p70S6K1 (AF6226), and phospho-p70S6K1 (AF3228) were all obtained from Affinity Biosciences (Jiangsu, China). An aliquot of 10 μg of tissue of cellular protein was subject to electrophoresis. The bound antibody was visualized with an enhanced chemiluminescencent detection kit (Yeasen, Shanghai, China). The bands for LAT1, VDR, and p70S6K1 were detected at 55KD, 48KD and 70KD, respectively. The band density was analyzed by ImageJ software (National Institutes of Health, USA). β-actin expression was determined and used to normalize relative protein expression in each sample.

### Immunofluorescent staining

HTR-8/SVneo cells were cultured on glass coverslips in 24-well plates (1 × 10^5^ cells/well) and fixed with ice-cold methanol and permeabilized with Triton X-100. Cells were incubated with primary anti-human LAT1 antibody followed by matched secondary antibody. After staining, coverslips were mounted on glass slides with 30% glycerol with 4,6-diamido-2-phenylindole (DAPI) and reviewed under a fluorescent microscope (Nikon corporation, Tokyo, Japan). Images were captured with a digital camera linked to a computer with imaging software (PreciPoint, Germany).

### Data presentation and statistics

All data are presented as mean ± SEM. Statistical analysis was performed with unpaired *t* test or One-way ANOVA using GraphPad Prism 8 software. A Tukey test was used as post hoc test. A value of *P* < 0.05 was considered statistically significant.

## Discussion

Emerging studies have shown that low maternal vitamin D levels in pregnancy was associated with increased risk of preeclampsia and preterm birth, suggesting that vitamin D deficiency is a risk factor of preeclampsia^23,24^. However, the underlying mechanisms remain unclear. In the current study, we demonstrate that placental LAT1 expression is reduced in women with preeclampsia, and 1,25(OH)_2_D_3_ stimulates LAT1 expression through VDR in cultured placental trophoblasts. Since altered placental transport of amino acids has been implicated in the fetal growth restriction in preeclampsia^4^, our findings suggest that the role for vitamin D in placental amino acid transporter is one possible mechanism underlying the linkage between maternal vitamin D levels and fetal growth in preeclampsia.

The fetal growth is largely dependent on placental transport of nutrients. The studies mentioned above and other studies have indicated that an altered placental transport of amino acids is intimately associated with fetal growth in pregnancy^4,5,25^, but controversial data are present regarding the modifications of placental amino acid transport in pregnancy disorders. For example, using IHC, Aiko et al. found that L-type amino acid transporter 4F2hc and LAT1 was increased in placental syncytiotrophoblast from pregnancies complicated by preeclampsia or intrauterine growth restriction (IUGR), suggesting an adaptive response to help maintaining growth in both of pregnancy complications^26^. Using real-time PCR, Malina et al. reported that mRNA levels for placental A-type amino acid transporters is not different between preeclampsia and normal pregnancies^27^. Interestingly, Shibata et al. found that A-type amino acid transporter activity was not reduced in the placentas of SGA infants from preeclamptic pregnancies, but significantly reduced in the placenta from SGA pregnancies without preeclampsia^28^, suggesting that growth restriction in the two disorders may be largely different. A recent study using multiple approaches including bioinformatic analysis, molecular biology, and mathematical diagramming demonstrated that amino acid transporter y^+^LAT1 was significantly increased in placentas associated with PE, but decreased in IUGR placentas^29^. This opposite but significant changes between preeclampsia and IUGR may suggest a different function of y^+^LAT1 in these two diseases. y^+^LAT1 is responsible for the transport of Na^+^-independent cationic amino acids, such as lysine, ornithine and arginine^30^. High expression of placental y^+^LAT1 could result lowered maternal levels of lysine, ornithine and arginine during pregnancy, which may elicit similar symptoms as reported in women complicated with lysinuric protein intolerance. Nevertheless, the pregnancy with lysinuric protein intolerance has been associated with an increased risk of serious complications, including preeclampsia^31^.

In the present study, the findings regarding the reduced placental LAT1 expression might be associated with fetal growth restriction observed in preeclampsia. LAT1 is a major Na^+^ independent transporter for indispensable amino acids, such as lysine, leucine, and histidine et al. Low expression of placental LAT1 associated decreased transport of amino acids across the placenta might cause high maternal plasma amino acid concentrations, which is in agreement with the previous findings as reported by Powers et al.^32^. The differentially expression patterns of placental LAT1 in preeclampsia in the above studies might result from the differing methods, sample size, or criteria of preeclampsia sample selection.

Information for the regulation of vitamin D on amino acid transport in placental trophoblasts is very scarce. Only Jansson et al. reported that bioactive 1,25-dihydroxy vitamin D_3_ markedly increased mRNA expression of the A-type isoform SNAT2 and the activity of A-type transporters in primary human placental trophoblasts, but had no effect on L-type and did not affect mTOR signaling^33^. On the contrary, our results showed that 1,25-dihydroxy vitamin D_3_ could significantly stimulate the protein expression of L-type transporter LAT1 under normoxic condition in cultured placental trophoblasts, and more importantly attenuate the decrease of LAT1 caused by CoCl_2_-induced hypoxia. We further demonstrated that knockdown of VDR by siRNA silencing prevented 1,25-dihydroxy vitamin D_3_-stimulated LAT1 expression in placental trophoblasts. Our findings suggest that vitamin D-stimulated LAT1 may make up for the shortage of decreased placental LAT1 in preeclampsia, which helps to maintain fetal growth. Those opposite findings between our and Jasson’s study indicate that the role for vitamin D-regulated LAT1 in preeclampsia should be further investigated.

Regarding the role for vitamin D in mTOR signaling, we found a similar trend as the vitamin D-regulated LAT1 that 1,25-dihydroxy vitamin D_3_ could prevent CoCl_2_-induced decrease of mTOR activity. However, 1,25-dihydroxy vitamin D_3_ had no effect on mTOR signaling in placental trophoblasts under normoxic condition, which was consistent with the results in Jasson’s study^33^. The VDR knockdown produced by siRNA silencing blocked the effects of vitamin D on CoCl_2_-induced mTOR activity. Since the mTOR signaling is directly implicated in the expression of LAT1 which is also confirmed in the present study, we propose that vitamin D-stimulated LAT1 might be mediated by mTOR signaling in placental trophoblasts. There was a study revealed that the role for vitamin D in mTOR signaling involved occupancy of vitamin D response elements (VDREs) of the gene for DNA-damage-inducible transcript 4 (DDIT4), an inhibitor of mTOR signaling^34^. They found that DDIT4 was a direct target for 1,25-dihydroxy vitamin D_3_ and could be induced by 1,25-dihydroxy vitamin D_3_ treatment in osteoblasts, which might be resulted from the competition for the binding to DDIT4 gene promoter by VDR and VDRE binding protein^34^.

In the past decade, several novel vitamin D metabolites including 20(OH)D_3_, 22(OH)D_3_, 20,22(OH)_2_D_3_ and 20,23(OH)_2_D_3_ were recognized in placenta, adrenal glands, and epidermal keratinocytes, suggesting 1,25(OH)_2_D_3_ is not solely bioactive form of vitamin D in vivo^35,36^. And their action may not through the genomic site of the VDR^37^. However, the biological functions of these vitamin D hydroxyderivatives in placenta and preeclampsia are still unknown (Supplementary Figures).

In summary, our study shows that placental LAT1 expression is reduced in preeclamptic pregnancies and vitamin D stimulated-LAT1 expression may be mediated by mTOR signaling in placental trophoblasts. Our results provide evidence for the vitamin D supplementation during pregnancy may be beneficial for reducing the risk of fetal growth restriction observed in preeclampsia probably by increasing essential amino acid transport across the placenta.

## Supplementary Information

Supplementary Figures.

## Data Availability

The datasets are available from the corresponding author on reasonable request.
