# Supplementary material for: Vitamin D stimulates placental L-type amino acid transporter 1 (LAT1) in preeclampsia
Source: Sci Rep. 2022 Mar 17;12:4651. doi: 10.1038/s41598-022-08641-y (PMC8931068; doi:10.1038/s41598-022-08641-y)
Supplement: Supplementary file 1 — Supplementary Figures. [file 41598_2022_8641_MOESM1_ESM.pdf]

Vitamin D stimulates placental L-type amino acid transporter 1 (LAT1) in preeclampsia

Xiaotong Jia[1#], Yang Cao[1#], Lingyu Ye[1], Xueqing Liu[1], Yujia Huang[1], Xiaolei Yuan[2], Chunmei Lu[1], Jie Xu[1*], Hui Zhu[1*]

1 Department of Physiology, Harbin Medical University, Harbin,150081, China

2 Department of Obstetrics & Gynecology, Second Affiliated Hospital of Harbin Medical University. Harbin,150081, China

[#] These authors contributed equally to this work

* Correspondence: xujie@ems.hrbmu.edu.cn; zhuhui@ems.hrbmu.edu.cn

Figure 1B

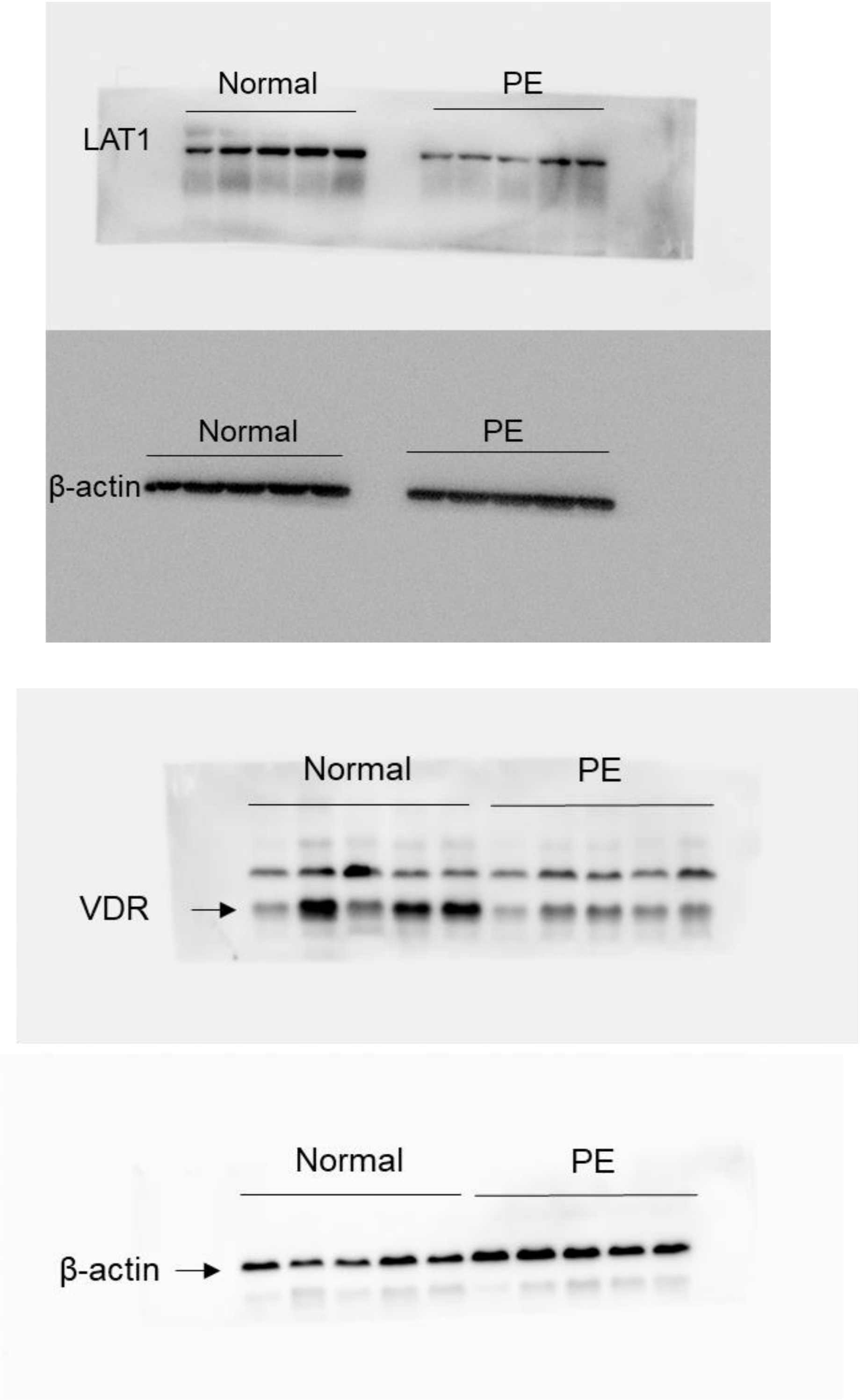
Normal
PE
LAT1
Normal
PE
β-actin
Normal
PE
VDR
Normal
PE
β-actin

Figure 2A

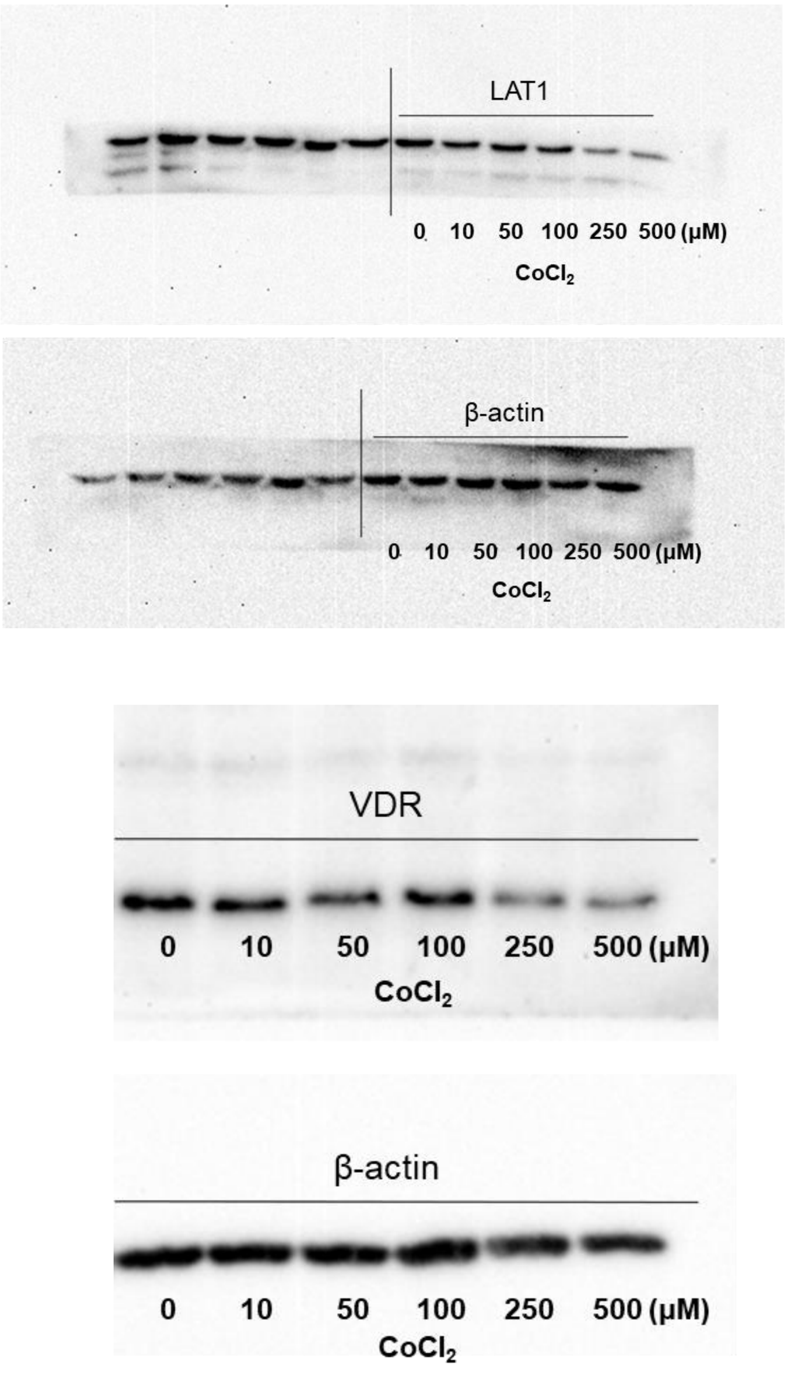

LAT1
0 10 50 100 250 500 (μM)
CoCl2
β-actin
0 10 50 100 250 500 (μM)
CoCl2
VDR
0 10 50 100 250 500 (μM)
CoCl2
β-actin
0 10 50 100 250 500 (μM)
CoCl2

Figure 2B

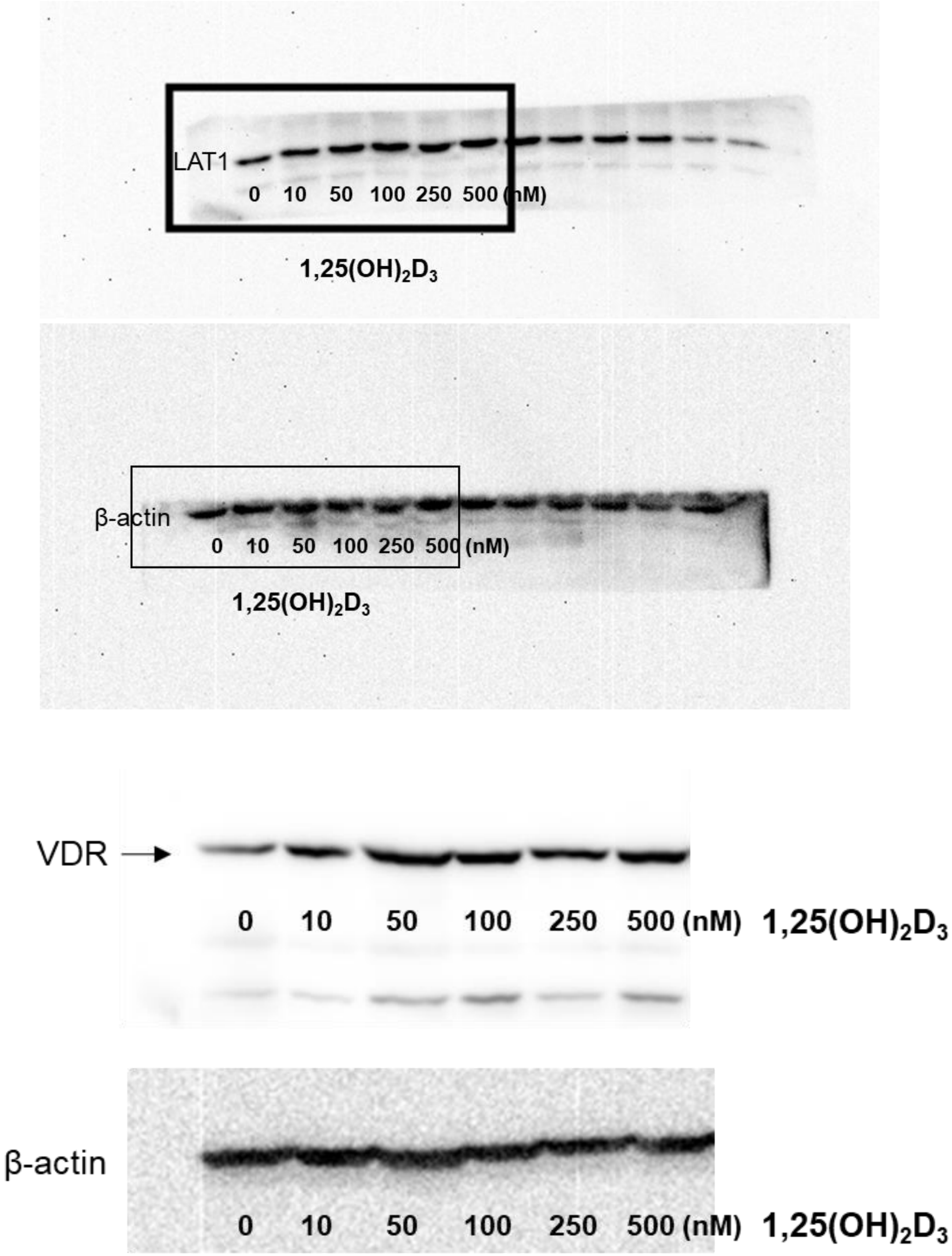
LAT1
0 10 50 100 250 500 (nM)
1,25(OH)2D3
β-actin
0 10 50 100 250 500 (nM)
1,25(OH)2D3
VDR
0 10 50 100 250 500 (nM) 1,25(OH)2D3
β-actin
0 10 50 100 250 500 (nM) 1,25(OH)2D3

Figure 3A

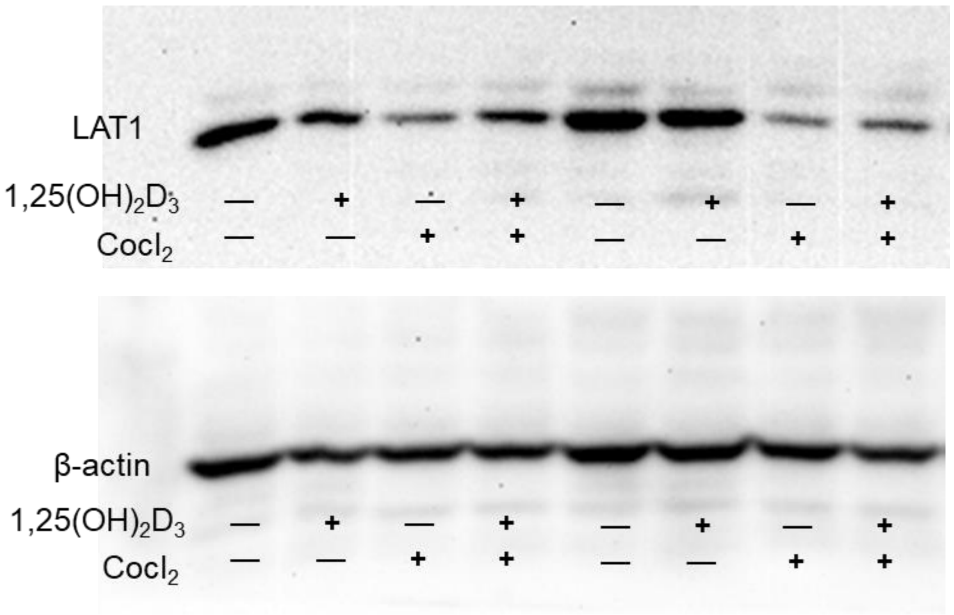

Figure 3C

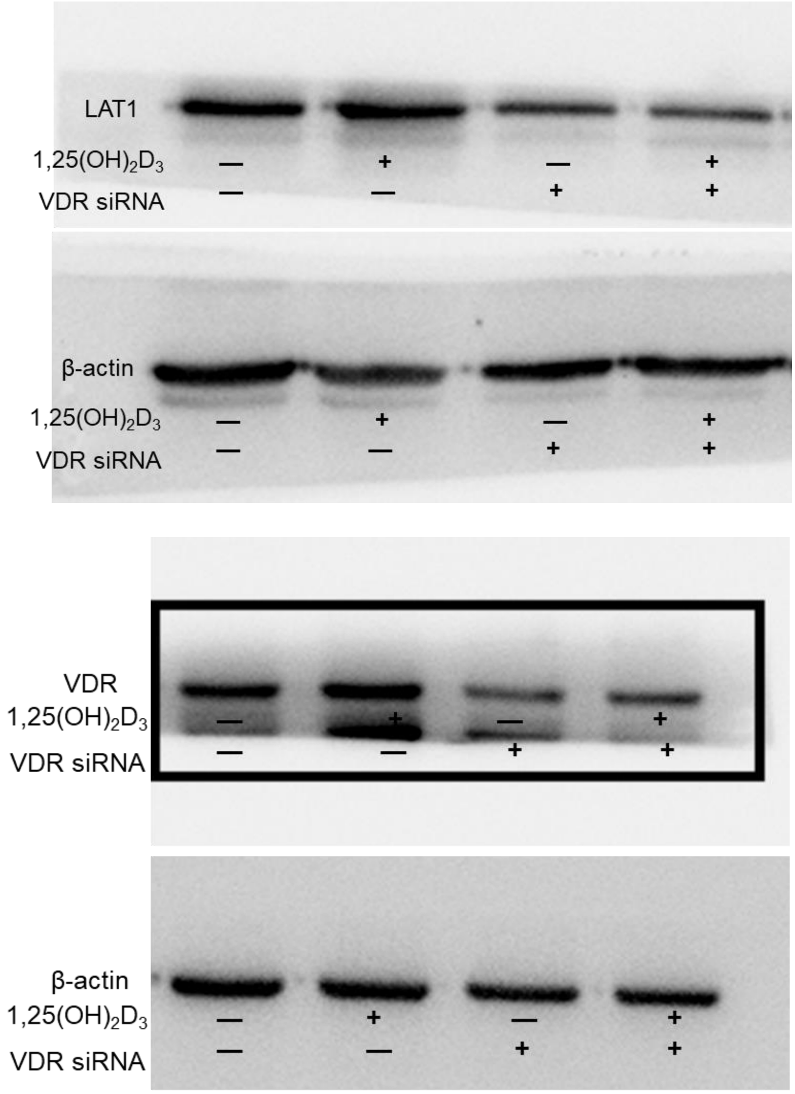

LAT1
1,25(OH)2D3 — + — +
VDR siRNA — — + +
β-actin
1,25(OH)2D3 — + — +
VDR siRNA — — + +
VDR
1,25(OH)2D3 — + — +
VDR siRNA — — + +
β-actin
1,25(OH)2D3 — + — +
VDR siRNA — — + +

Figure 4A

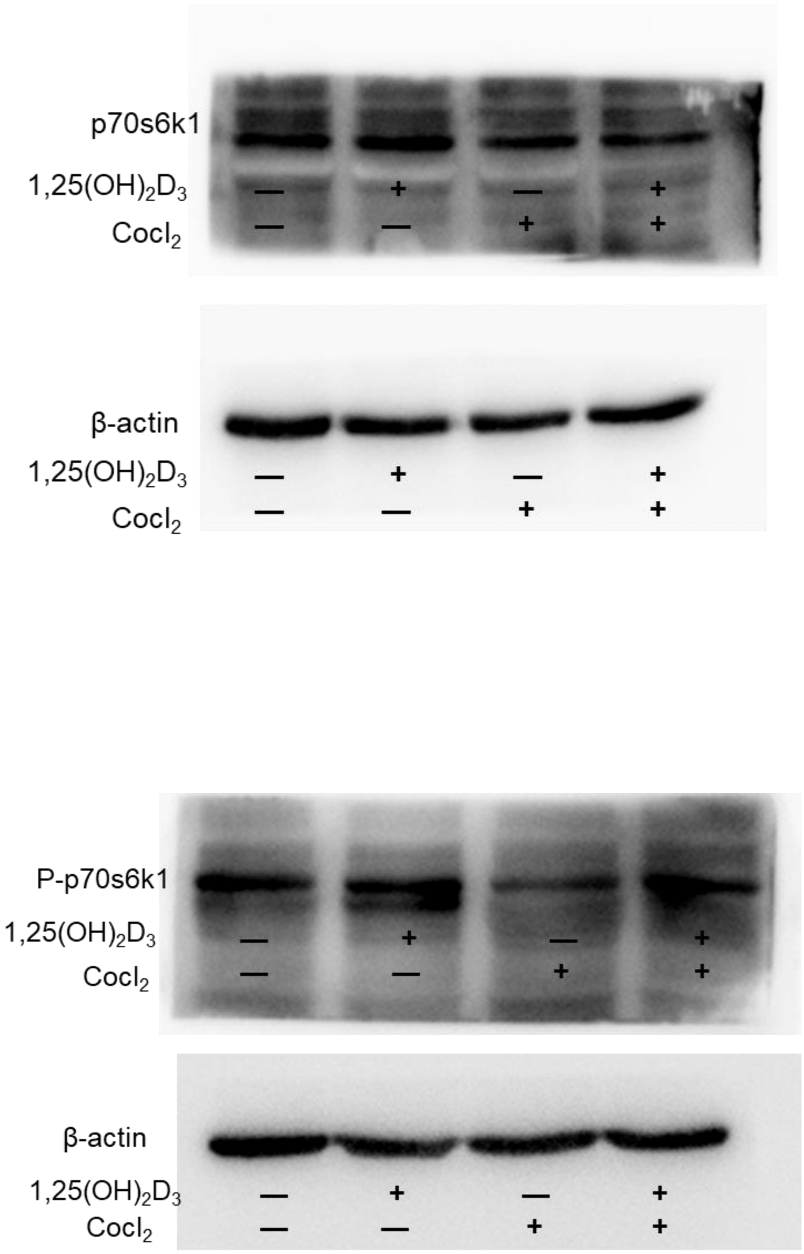

p70s6k1
1,25(OH)2D3 — + — +
Cocl2 — — + +
β-actin
1,25(OH)2D3 — + — +
Cocl2 — — + +
P-p70s6k1
1,25(OH)2D3 — + — +
Cocl2 — — + +
β-actin
1,25(OH)2D3 — + — +
Cocl2 — — + +

Figure 4B

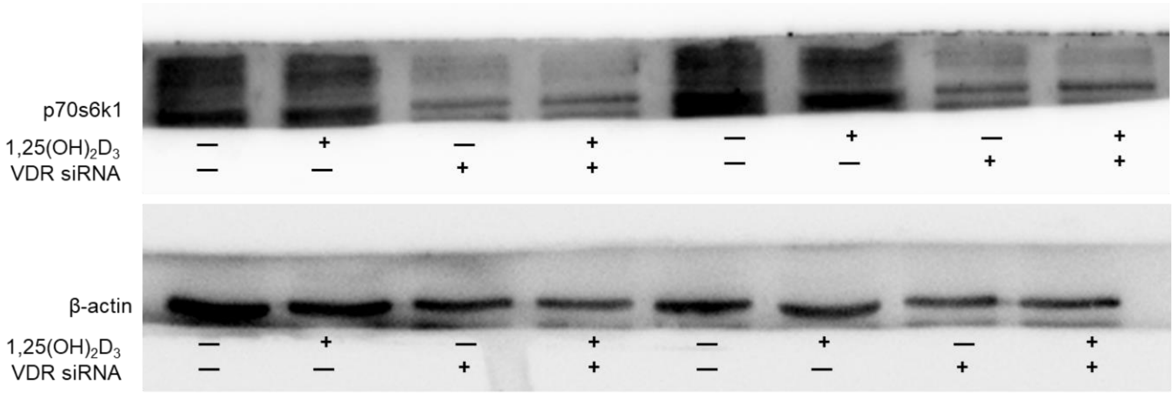
p70s6k1
1,25(OH)2D3
VDR siRNA
β-actin
1,25(OH)2D3
VDR siRNA

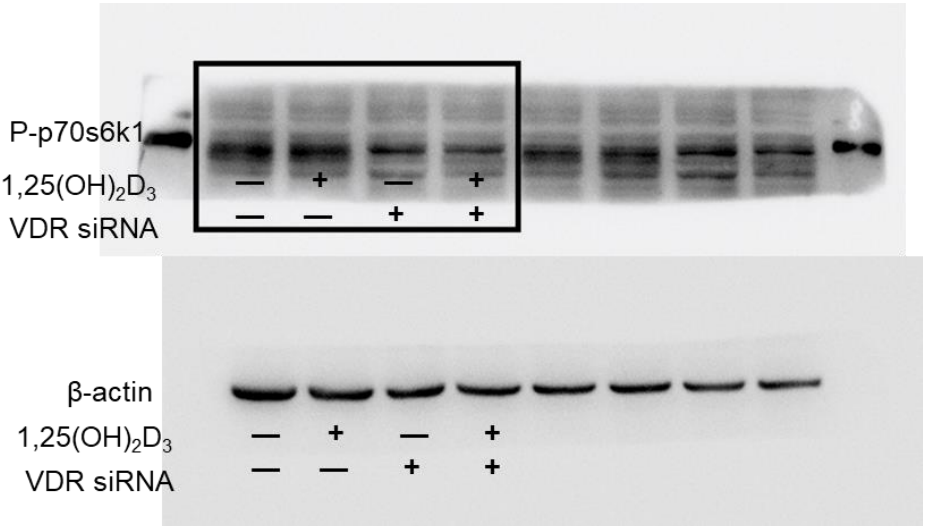
P-p70s6k1
1,25(OH)2D3
VDR siRNA
β-actin
1,25(OH)2D3
VDR siRNA

Figure 5A

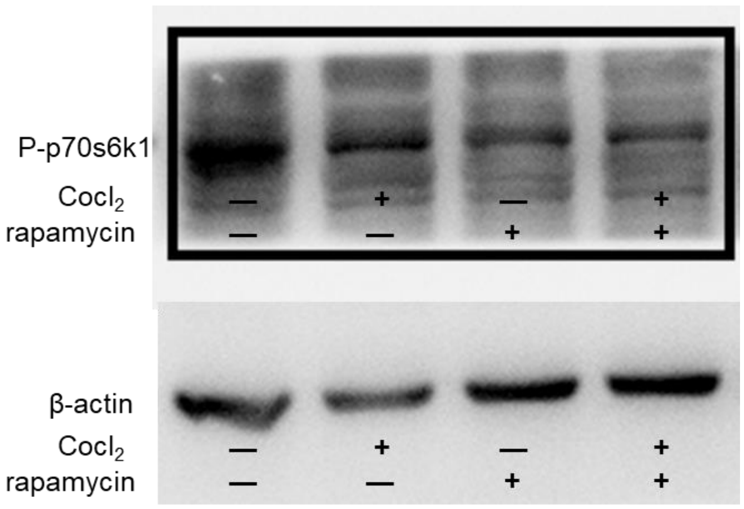
P-p70s6k1
$CoCl_2$ — + — +
rapamycin — — + +
β-actin
$CoCl_2$ — + — +
rapamycin — — + +

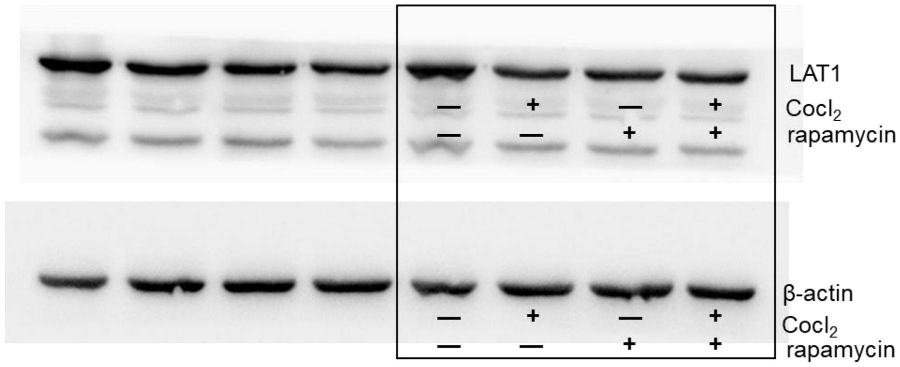
LAT1
$CoCl_2$ — + — +
rapamycin — — + +
β-actin
$CoCl_2$ — + — +
rapamycin — — + +
